# Malaria transmission in non-endemic areas: case report, review of the literature and implications for public health management

**DOI:** 10.1186/1475-2875-8-71

**Published:** 2009-04-20

**Authors:** Thomas Zoller, Torsten J Naucke, Jürgen May, Bodo Hoffmeister, Holger Flick, Christopher J Williams, Christina Frank, Frank Bergmann, Norbert Suttorp, Frank P Mockenhaupt

**Affiliations:** 1 Medizinische Klinik mit Schwerpunkt Infektiologie und Pneumologie, Charité – Universitätsmedizin Berlin, Augustenburger Platz 1, 13353 Berlin, Germany; 2 Institut für Medizinische Mikrobiologie, Immunologie und Parasitologie (IMMIP), Universitätsklinikum Bonn, Sigmund Freud Straße 25, 53105 Bonn, Germany; 3 Institut für Zoologie, Fachgruppe Parasitologie, Universität Hohenheim, 70599 Stuttgart, Germany; 4 Bernhard Nocht Institute for Tropical Medicine, Infectious Disease Epidemiology, Bernhard-Nocht-Straße 74, 20359 Hamburg, Germany; 5 Robert-Koch-Institut, Department for Infectious Disease Epidemiology, Seestraße 10, 13353 Berlin, Germany; 6 Institute of Tropical Medicine and International Health, Charité – University Medicine Berlin, Spandauer Damm 130, 14050 Berlin, Germany

## Abstract

In non-endemic areas, malaria is rare and locally acquired infections, particularly with *Plasmodium falciparum*, are exceptional events. The diagnosis is, therefore, likely to be delayed or missed in patients without a relevant travel history. This report describes a case of falciparum malaria in Berlin, Germany, in a patient who had not been to an endemic area for more than a decade. Potential routes of vector-related and direct transmission were evaluated, particularly with regard to a possible danger to the public. A review of the literature was conducted regarding possible routes of transmission and their probability assessed. Genotyping of parasite isolates of this and another patient with malaria admitted 16 days before revealed homology between the two strains. In a local entomological survey, anopheline vectors on the hospital grounds as well as in the residential area of both patients were found. Despite intensive investigations, the mode of transmission remained obscure. In this context, possible routes of vector-borne and direct occupational/accidental transmission in a major European city are reviewed and discussed, providing information and guidance in case other similar events occur elsewhere. Examples for investigations and measures to be taken in such a situation are provided. When local malaria transmission within a large non-immune population cannot be ruled out, genotyping of parasite isolates, local entomological surveys, preparedness for secondary cases, expert consultations in a multidisciplinary team and careful information management are essential. Malaria acquired in non-endemic areas remains an unlikely, but possible event for which awareness needs to be maintained.

## Background

Transmission of malaria in a non-endemic area is an extremely unusual event, but is possible under certain conditions. For those affected, the combination of a missed or delayed diagnosis along with the high fatality of *Plasmodium falciparum *infection may result in serious consequences. These rare occasions pose a challenge to local and public health authorities.

In the European Union, 6,000–10,000 imported cases of malaria and 26 to 78 attributable deaths have been reported annually between 1994 and 2006 [1]. In sick travellers returning from malaria-endemic areas, a detailed travel history is mandatory and malaria must be excluded. *Plasmodium falciparum-*infected individuals without a relevant travel history are at high risk of a delayed/missed diagnosis and, consequently, of developing potentially life-threatening severe falciparum malaria.

Local transmission leading to isolated malaria cases in non-endemic regions has occurred under various circumstances including (i) "airport", (ii) "port", and (iii) "baggage" malaria, (iv) nosocomial transmission and (v) transmission by local competent vectors. Also, small malaria epidemics with continued localized transmission occurred in northern Germany and Berlin up to 1947 [2], and, more recently, in the USA [3].

With the aim to raise awareness for the potential of locally acquired malaria and to provide information and guidance, this report discusses possible routes of vector-borne and occupational/accidental transmission. As an example for investigations and measures to be taken in such a situation, a case of local transmission of *P. falciparum *in Berlin, Germany, which occurred in July 2007 is presented.

## Case presentation

A consultant physician of the Department of Obstetrics & Gynaecology presented to the emergency room of the same university hospital in Berlin on 26 July 2007. She reported a four-day history of fever and headache. Common causes of fever were ruled out. On questioning, the patient stated that she had never been to Asia or Africa. However, she had travelled to Colombia eleven years before where she contracted malaria. Despite an extremely low probability of a tertian malaria relapse after this time, thick and thin blood films were examined, revealing *Plasmodium falciparum *at a density of 0.5% parasitized erythrocytes. *Plasmodium falciparum *mono-infection was confirmed by PCR [4]. Other laboratory abnormalities included a thrombocytopaenia of 50/nL, an elevated C-reactive protein of 9.74 mg/dL, and leukocyturia. The patient was treated with oral artemether/lumefantrine and recovered fully within three days.

**Figure 1:**
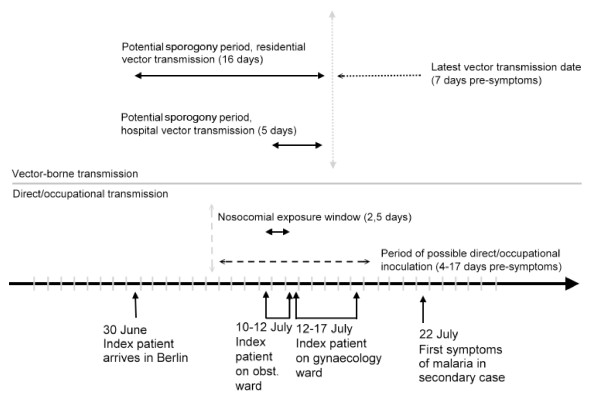
**Timeline of events**. *Vector-borne transmission *(upper part): theoretical minimum time for sporogony is 10 days under African temperature conditions. Under temperatures in Berlin during this time, at least 21 to 24 days can be expected (see text for details) – sporogony periods for both hospital and residential vector transmission are too short, arguing against this possibility of transmission. *Direct/occupational exposure *(lower part): only during the stay of the index patient on the obstetrics ward, there was a theoretical possibility of direct inoculation of infectious material, a short incubation time of 4 up to17 days can be expected. Occurrence of symptoms on 22 July was within the time frame to be expected for this scenario.

Because the travel history was not consistent with imported malaria, an investigation for a local source of infection was carried out. A pregnant patient with falciparum malaria (hereafter referred to as the "primary case") had been admitted to the delivery room on 10 July 2007, 16 days before the consultant was diagnosed with malaria. The primary case had returned to Berlin from a four-weeks visit to the Ivory Coast on 30 June 2007. She had taken no chemoprophylaxis. Ivorian born, she had been a Berlin resident for 7 years. After returning to Berlin, she developed fever on 8 July 2007. On admission, she presented with a *P. falciparum *mono-infection (PCR confirmed) with 3% parasitaemia. She was treated with mefloquine, recovered fully and was discharged from hospital on 16 July 2007. For a timeline of events, see Figure 1.

The consultant ("secondary case") encountered the primary case in person on only two occasions, once in the evening of admission to the labour ward (10 July) and then again the following morning. The secondary case, supervising the resident physicians, could not recall any direct physical contact on these two occasions, apart from possibly shaking hands. In particular, the consultant did not perform any medical procedures on the primary case at any time during the entire hospital stay, nor did she recall any needlestick injuries. On one occasion, the consultant hand-carried a fully bagged blood sample of the primary case, which had been collected by another physician on admission; she could, however, not recall seeing any blood on the outside of the bag.

### Methods and results regarding possible modes of transmission and management

Suspecting local malaria transmission, all possible routes of transmission, as outlined above, had to be considered at the outset. One scenario included a possible danger to the public from infective mosquito(es) present on the hospital compound or in the residential area where both cases live.

### Genetic homology of parasite strains

Parasite isolates from primary and secondary case were genotyped for the highly polymorphic *P. falciparum msp-1 *and *msp-2 *genes [5]. The results revealed complete homology. This was confirmed in a second laboratory with a second set of samples and by extending genotyping to PfRRM microsatellite markers [6] and polymorphic DBL-alpha sequences [7]. In contrast, human microsatellite markers showed dissimilarity between the two samples, excluding a mix-up or contamination of the samples taken from both cases [6].

### Patient interviews

#### Sources of occupational or accidental inoculation

The delivery room and maternity wards were thoroughly inspected and all persons directly or indirectly involved in the management of the primary case were repeatedly interviewed. No exposures compatible with a nosocomial transmission could be recalled. In particular, the absence of physical contact between index and secondary case was confirmed independently. The secondary case did not recall any injections, accidents or blood transfusions in the months preceding the malaria episode. She had no relevant skin damage during the possible direct-transmission window, apart from a small dressed wound on the wrist following a horsefly bite. Moreover, she stated that this wound had begun to close by the 10^th ^July 2007. She could not recall changing the dressing whilst on the wards.

#### Baggage malaria

The index patient had used the same baggage both for her trip to the Ivory Coast and her hospital admission 11 days after returning. Both index patient and her husband stated that the bags had been completely emptied after returning to Berlin, and on inspection, nothing unusual was observed.

#### Contact or transmission in residential area

Further interviews with the two patients revealed that both live in the same, densely populated residential area of Berlin, approximately 1.8 km apart from each other. However, none of the places frequently visited by the patients in their daily activities were common to both.

### Entomological survey

In August 2007, an entomological survey on the hospital compound and the patients' area of residence was carried out. In both places, viable *Anopheles *larvae were found: on the hospital compound in small quantities in a fountain, and in the residential area in a backyard water butt. Morphological examination of the Anopheles larvae identified them as the species-complex *Anopheles maculipennis s.l*., which includes species with potential vectorial competence [8,9]. Adult *Anopheles *mosquitoes were not found.

### Information management of the suspected outbreak situation

Meanwhile, the federal health administration of Berlin convened a panel, which included infectious disease and public health specialists, along with communications officers. Although the occurrence of other mosquito-transmitted secondary cases seemed unlikely, this risk could not completely be ruled out at the time of the investigation. A selective approach to risk communication was used, with an alert sent only to hospitals in Berlin with emergency departments. The alert advised clinicians to consider malaria in cases of unexplained fever, particularly when coinciding with thrombocytopaenia. No further, presumably locally acquired, *P. falciparum *infections were reported to the health authorities. Public media did not report the incident.

## Discussion

Malaria has been endemic in Europe and particularly around the North Sea at least from the 7^th ^century until the late 1950s [10]. Martini [11] reported an epidemic on the German North Sea coast in 1826 with several thousand cases and many casualties; transmission was facilitated by unusually high summer temperatures. The last reported cases of autochthonous tertian malaria were observed in Berlin and Hamburg in 1947 [2]. *Plasmodium falciparum *is thought to have never been endemic in Germany, but continues to be imported by travellers returning from endemic areas.

In the last six decades, only three cases of potentially local *P. falciparum *transmission have been reported from Germany. One of these, involving presumed transmission by local *Anopheles plumbeus *on a hospital compound in Duisburg, Western Germany, shows some parallels to the case reported here [12]. Two simultaneous cases of supposed mosquito-borne infection occurred in 1994 close to a sewage plant in Berlin [13]. In the present case, genetic analysis of parasite isolates did confirm the index patient as the source of the infection in the secondary patient.

### Requirements for local mosquito transmission

Larvae of *A. maculipennis s.l*. were identified both on the hospital compound and in the patients' residential area. The time period required for completion of sporogony (extrinsic incubation time) is critical in determining the likelihood of mosquito-borne transmission. This period is highly temperature-dependent, but usually takes at least seven days under optimal average ambient temperatures in Africa of 35°C [14] and up to 30 days under ambient temperatures of 20°C [2]. Mosquitoes of the *A. maculipennis*-species complex are potential vectors of *P. falciparum *[15].

### Transmission by local Anopheles mosquitoes on the hospital compound

In Berlin, temperatures were exceptionally high exactly during the time of potential extrinsic incubation with night minimum temperatures >14°C in two nights and >17°C in the following five nights. Local temperature data was analysed and a probable extrinsic incubation time of 21 to 24 days calculated for *P. falciparum *[2]. Mosquitoes resting within buildings (e.g. in the basement) may have been exposed to higher average temperatures. Adding to the extrinsic incubation period, the minimum intrinsic incubation time of *P. falciparum *infection in human experimental malaria is estimated as seven days [16]. Assuming local transmission by anopheline vectors, the entire incubation period in this case (extrinsic and intrinsic) would amount to a minimum of 28 days, based on theoretical considerations using experimental data currently available. This, however, exceeds the timespan of 11 days between the index patient's admission and first symptoms in the secondary case (see Figure 1) arguing against a mosquito-borne transmission on the hospital grounds.

### Transmission by local Anopheles in the residential area

The index and the secondary patient live in the same centrally located borough of Berlin, some 1.8 km apart. After returning from Ivory Coast on 30 June, the index patient stayed in this area for eleven days before being admitted to hospital. Theoretically, intrinsic *plus *extrinsic incubation period and the presence of *An. maculipennis s.l*. would allow a mosquito-borne transmission of *P. falciparum *in the city. However, it would be unusual, though not impossible, for a local anopheline species to travel more than 2 km within a short period of time. Furthermore, it seems very improbable that this unlikely event (of transmission in the residential area) should be followed by the even less probable coincidental meeting of index and secondary case in the same hospital as doctor and patient.

Eventually, genetic analysis showed a high degree of homology between both parasite isolates. Although this does not completely exclude a mosquito passage of *P. falciparum *(in which sexual parasite reproduction and genetic recombination occur), it renders both scenarios involving vector-borne transmission unlikely.

### Airport/port malaria

"Airport malaria" results from infective bites of mosquitoes, which travelled aboard an airplane and has occurred in a number of instances around major airports throughout Europe. Mosquitoes can survive a flight even in non-pressurized wheel bays [17]. In a series of 29 respective patients from 1969 to 1988, most cases occurred within a distance of 2 km from the next airport, but longer distances are also possible when vectors are transported further, for example in a car [18]. In this series, four patients died due to a delayed or missed diagnosis and approximately one third of the cases were persons working at or around the airport. A case of "port" malaria has been observed about 3.5 km off the port of Ghent, Belgium [19]. In the secondary malaria patient, an "airport" malaria was considered due to a recent visit to Frankfurt airport, but dismissed after genotyping results revealed homology with the index patient's parasite strain.

### Baggage malaria

The possibility of carrying infective mosquitoes within pieces of baggage to non-endemic areas or to areas far from international airports has been suspected in a number of previous cases [13,20,21]. The homology of strains in the two patients could accord with transmission by the same mosquito. In this scenario, the mosquito was carried in the baggage from Ivory Coast to Berlin, infected the index case before or during travel or at home in Berlin and then – after being transported to the hospital – infected the secondary patient.

### Nosocomial transmission

Nosocomial *P. falciparum *infection, in which blood or parasite-containing fluids from a parasitaemic patient enter the bloodstream of a secondary patient, are well-documented. Example routes include needlestick injuries [22-26], incorrect use of blood glucose meters [27], or failure to exchange needles using multidose drug vials [28-30]. The incubation time following direct blood contact or needlestick injuries ranges from 4 to 17 days (median 12 days) [31].

There is also evidence of transmission through contaminated surfaces. This has been implicated in cases of malaria following re-use of catheters for contrast media [32] or handling of intravenous lines with contaminated gloves [33] and suggests that the infection can be passed by an intermediate and not necessarily visibly blood contaminated object. However, transmission in the absence of percutaneous or intravenous breaches has been described only in cases of direct contact with visible and infected blood. Published examples include a nurse with fingertip skin scratches (caused by peeling potatoes) [34], and a doctor with a minor nail-cutting injury; both became infected after having venosected malaria patients [35]. In the broader nosocomial context, self-inoculation of patients' blood as a consequence of a psychiatric disorder has been reported from a German hospital, producing a series of thirteen malaria episodes in a laboratory technician [36].

The secondary case denied any needlestick injuries, invasive medical procedures performed on herself, or any contact between skin breaks and visible blood or contaminated fluids from the index case. However, her recall may not have been perfect, as she was interviewed nearly a month after the possible exposure time.

### Probable route of transmission

In the present case of falciparum malaria acquired locally in Berlin, Germany, the route of transmission remains obscure despite extensive investigations. Transmission by local *Anopheles *on the hospital compound seems improbable in view of the timeline. While incubation times would allow for mosquito-borne transmission in the residential area, this option would require combinations of *per se *unlikely events. In addition, the absence of genetic recombination renders a mosquito-borne transmission unlikely. "Baggage malaria" cannot be ruled out but also appears unlikely: the mosquito would have had to survive for 11 days in the flat in Berlin (all bags had been emptied), re-enter the baggage in which it is was imported from Ivory Coast, to be carried to the hospital where it infected the secondary case. Nosocomial transmission would require fewer improbable events but suffers from the lack of a clear exposure history for the secondary case.

### Implications for public health management

Because of the high fatality of undiagnosed falciparum malaria, suspected mosquito-borne transmission in non-endemic areas has significant public health implications. Protecting the population, even against a hypothetical threat, has highest priority under such circumstances.

In the case described here, mosquito-borne transmission was assumed in the early stages of investigation. It had to be considered that at least one infectious *A. maculipennis s.l*. could pose a danger during the rest of its lifespan (approximately six weeks, < 1 blood meal/week). In case of "baggage malaria", *Anopheles gambiae *would have been present for at least a few days on the hospital compound (life span approximately three weeks, one blood meal every other day). Careful information management and adequate risk communication in such a case is essential: missing potential further cases of autochthonous malaria must be avoided, but the extensive media coverage that might have followed a wider alert was also undesirable. In particular, with extensive media coverage, a high number of febrile patients with other diagnosis would need to undergo malaria testing. Here, the policy of selectively informing key health facilities and physicians who have initial contact with febrile patients proved to be adequate. In parallel, preparedness for potential subsequent events included surplus procurement of rapid test devices, allocation of further diagnostic capacity (staff, infrastructure), daily exchange of information between stakeholders, and training of personnel primarily involved in case of upward disease activity. Generally, in places outside endemic areas but with temporal presence of vectors competent for transmitting malaria, artemisinin-based anti-malarial therapy may contribute to reduce the risk of local transmission due to the gametocidal activity of artemisinins [37].

When malaria transmission within a large non-immune population cannot be ruled out, a case definition is required to determine which patients should undergo testing for malaria. The most common clinical symptoms of *P. falciparum *malaria among returning travellers are fever (93%), headache (51%), musculoskeletal pain (35%) and fatigue (32%) [38]. Suggestive biochemical markers involve (i) thrombocytopaenia, (ii) elevation of serum lactate dehydrogenase and (iii) anaemia. Among more than 4,000 unselected patients in Lisbon, Portugal, seeking care in a hospital emergency department for any reason, the prevalence of thrombocytopenia of less than 150,000/μL, 100,000/μL, 50,000/μL was 7%, 2.2% and 0.6%, respectively. Screening all samples with a thrombocyte count <100,000/μL led to the detection of five unsuspected malaria cases [39]. Of emergency department patients in Lisbon and Berlin in whom malaria was clinically suspected and who presented platelet counts <150.000/μL, more than 75% were found to be positive for malaria [39]. In a non-endemic area with a susceptible population, a pragmatic approach to cope with hypothetical *P. falciparum *transmission could, therefore, be to limit malaria diagnostic testing to selected individuals, such as those presenting with at least one malaria-suggestive symptom and a platelet count below 150,000/μl. From the experience with the reported, probable case of locally acquired malaria, a set of measures outlined in Table 1 is recommended.

**Table 1:** Key recommendations for public health management in malaria infections outside endemic areas

• Rapid and regular assessment of new information by a team of clinicians, malaria experts, entomologists and public health experts
• Timeline of events with incubation times for different transmission scenarios to assess their probability

• Molecular genotyping of isolates from index- and secondary cases

• Repeated interviews of index- and secondary cases as well as all other personnel involved

• Local entomological survey

• Selectively informing key health infrastructures and physicians

• Preparation of a press release in case of public media attention

## Conclusion

In conclusion, the route of transmission in this unusual case of falciparum malaria in Berlin could not be determined. With climate change improving conditions for competent anopheline vectors in non-endemic areas, the risk of isolated *P. falciparum *infections or small outbreaks secondary to imported cases increases. This possibility should specifically be considered in large cities with high numbers of returning travellers and semi-immune migrants, who may carry infective gametocytes for extended periods of time. Due to its gametocidal activity, artemisinin-based anti-malarial therapy should be preferred in all settings with a competent vector population. Direct routes of transmission, such as nosocomial transmission or, rarely, self-inoculation should be considered in cases of otherwise unexpected *P. falciparum *infection. Genetic comparison of malaria species can be helpful in evaluating transmission scenarios. Even in non-endemic areas and in the absence of a travel history, it is important to consider malaria in patients with unexplained fever.

## Consent

Written informed consent was obtained from the patient for publication of this case report and any accompanying images. A copy of the written consent is available for review by the Editor-in-Chief of this journal.

## Competing interests

The authors declare that they have no competing interests.

## Authors' contributions

All authors have made substantial contributions to the investigations presented in this manuscript. TZ and FPM drafted the manuscript and all authors and have been involved revising it. All authors have read and approved the final version of the manuscript.
